# Prospect of nanomaterials as antimicrobial and antiviral regimen

**DOI:** 10.3934/microbiol.2023024

**Published:** 2023-05-10

**Authors:** Ashok Chakraborty, Anil Diwan, Jayant Tatake

**Affiliations:** AllExcel, Inc, Shelton, CT 06484, USA

**Keywords:** nanotechnology, nanovehicles, nanoparticles, nanocomposites, nanopolymer, nanoviricide, antimicrobial, antiviral, TheraCour, SARS-CoV-2

## Abstract

In recent years studies of nanomaterials have been explored in the field of microbiology due to the increasing evidence of antibiotic resistance. Nanomaterials could be inorganic or organic, and they may be synthesized from natural products from plant or animal origin. The therapeutic applications of nano-materials are wide, from diagnosis of disease to targeted delivery of drugs. Broad-spectrum antiviral and antimicrobial activities of nanoparticles are also well evident. The ratio of nanoparticles surface area to their volume is high and that allows them to be an advantageous vehicle of drugs in many respects. Effective uses of various materials for the synthesis of nanoparticles impart much specificity in them to meet the requirements of specific therapeutic strategies. The potential therapeutic use of nanoparticles and their mechanisms of action against infections from bacteria, fungi and viruses were the focus of this review. Further, their potential advantages, drawbacks, limitations and side effects are also included here. Researchers are characterizing the exposure pathways of nano-medicines that may cause serious toxicity to the subjects or the environment. Indeed, societal ethical issues in using nano-medicines pose a serious question to scientists beyond anything.

## 1. Introduction

Infectious disease, in recent times, is a great concern in public health. Around the world, microbial infection causes mortality in millions of people every year [1]–[4]. Further, the microbes can turn resistant to antibiotics due to their high mutative capacity and morphological changes [5]–[7]. A nanoparticle (NP)-based treatment approach could be promising to overcome the drug-resistant effects of the microbes. Further, NPs can have innate antimicrobial activities [8],[9]. NPs can generate reactive oxygen species (ROS), which can damage DNA and proteins and block the growth of bacteria, fungi and viruses. Antibiotics conjugated with nanoparticles have therefore been thought to be an efficient antimicrobial regimen [10]. The antibiotic cefaclor attached to gold nanoparticles (52–22 nm) showed significant antibacterial activity [11],[12]. Biogenic selenium nanoparticles have anti-biofilm activity and effectively retard the growth of *Pseudomonas aeruginosa*, a Gram-negative bacteria [13],[14]. Similarly, TiO_2_ nanoparticles have been found to inhibit the formation of fungal biofilms [15].

Nanotechnology can help the world's medical community to fight against virus infection also [16],[17]. For example, studies have been done successfully on the effects of nano-materials as antivirals against the virus SARS-CoV-2, inhibiting its entry into cells, its RNA replication and, finally, its release [18]–[20]. In addition, nano-materials provide a wide range of opportunities for diagnosis, treatment and in controlling the biofilm formation. Recent advances of applications of various nanomaterials in the diagnosis and treatments of microbial infections have been reviewed elsewhere [21]–[24]. However, their impact on human tissues and the environment should be assessed before implementations in large-scale industry are carried out [25].

Here, we discuss several aspects of using nanoparticles in infectious diseases, their pros and cons, challenges for nanoparticles and future prospects.

## 2. Nanoparticles/Nanocomposites and their antimicrobial properties

Nanoparticles (NPs) belong to a group of substances having diameters ranging from 1–100 nm [26]–[29], and they possess the ability to penetrate the bacterial cell wall, which is made up of peptidoglycan. NPs can dismantle the peptidoglycan layer from Gram-positive bacteria and also overcome antimicrobial resistance [5],[30],[31],

### 2.1. NPs and their antibacterial activities (Table 1)

**Table 1.** Antiviral nanoparticles and antibacterial activities.

Antibacterial Nanoparticles	Functions
Gentamicin coated phosphatidylcholine–chitosan hybrid nanoparticles [32]	Inhibit the growth of Gram-positive and Gram-negative bacteria [32]
Supramolecular polyelectrolyte complexes, (like NH_3_^+^ of the β-cyclodextrin-chitosan complexes with the negatively-charged SO_3_^−^ groups) [33].	Silver sulfadiazine molecules complexed with β-cyclodextrin releases silver ions which damages the bacterial cell wall [33]
Vancomycin antibiotic encapsulated in polymersomes [34]	Antibacterial effects against methicillin-resistant *S. aureus* [34]
Mannose-chitosan complex nanoparticles [35]	Mannose-chitosan complex nanoparticles have antibacterial activities against gram-positive and gram-negative bacteria [35]
Teicoplanin-containing polylactic-*co*-glycolic acid (PLGA) nanoparticles [36]	Showed an antibacterial effect on *S. aureus* [36]
*Pistacia lentiscus L. var. chia essential oil* can be encapsulated within PLA nanoparticles [37]	Showed an inhibitory effect on gram-positive and gram-negative bacteria [37]
Silver nanoparticles with PLA nanocoatings and with polyethylene terephthalate nanofibers [38]	Works against gram-positive and gram-negative bacteria, both [38]

### 2.2. NPs as an antiviral regimen (Table 2)

Viruses can infect prokaryotes as well as eukaryotes. Vaccines are effective in some of viral diseases such as smallpox, polio, etc, yet further opportunities to overcome antiviral drug resistance is possible by using NPs [39],[40].

**Table 2.** Antiviral nanoparticles and their functions.

Antiviral Nanoparticles	Functions
Chitosan nanoparticles complex with peptides derived from HIV-1 P24 protein [41].	Showed reduced toxicity and sustained peptide drug release [41].
NPs attached with hydroxypropyl-β-cyclodextrin and loaded with Dolutegravir [42].	Results in improved permeation of the drug through nasal mucosa without damaging the mucosa [42].

### 2.3. Application of NPs in fungal and parasite infections (Tables 3 and 4)

**Table 3.** Antifungal nanoparticles and their functions.

Antifungal Nanoparticles	Functions
Administration of miconazole and farnesol together with chitosan NPs [43]	The minimum inhibitory concentration (MIC) of nanosystems against *C. albicans* is similar to the values for the miconazole free drug [43]
Chitosan nanoparticles incorporating itraconazole [44]	Potentially inhibits the growth of *C. neoformans*, *C. albicans* and *A. fumigatus* [44]
Nanocapsules containing modified polysaccharide for the delivery of amphotericin B [45]	This nanosystem showed significant antifungal activity against *C. albicans* strains, compared to the free drug [45]

**Table 4.** Antiparasitic nanoparticles and their functions.

Antiparasitic Nanoparticles	Functions
A poorly water-soluble compound, Triclabendazole, encapsulated within chitosan [46]–[49]	Found successful in treatment of fascioliasis [46]
	Showed an inhibitory effect on *Leishmania* promastigotes protozoan parasites [47]

Some industrial and biomedical applications of nano-materials as alternatives to commercially available antibiotics and anti-fungal medications are reviewed in [22],[24] (Tables 5 and 6).

**Table 5.** Nanomaterials with antifungal activities.

Targets	Antifungal activity	Nanoparticle type	Route of administration	References
*Trichophyton rubrum*	AmB, CLT	SLN, SLN	Topical	[50],[51]
*Candida albicans*	CLT, ECN, MN	SLN-based stearate, SLN, SLN	Topical	[52]–[54]
*Candida species*	MN	SLN-bearing Hydrogel, SLN	Topical, Oro mucosal	[55],[56]
*Aspergillus flavus*	ITZ; VRZ	SLN	Ocular	[57],[58]
*Candida glabrata*	VRZ	SLN	Ocular	[59]
*Candida species*	FLZ	SLN	Topical	[60]
*Dermatophyte*	GF	SLN	N/A	[61]
*Candida tropicalis*	AmB	Ag	N/A	[62]
*Aspergillus niger*	AmB	Ag	N/A	[63]
*Fusarium culmorum*	AmB	Ag	N/A	[63]
*Aspergillus brasiliensis*	NYS, FLZ	Ag	N/A	[64]
*Malassezia furfur*	KTZ	Ag	Topical	[65]
*Paracoccidioides brasiliensis*	AmB	PLGA	N/A	[66]
*Candida parapsilosis*	AmB	CS-coated PCL	Oral	[67]
*Aspergillus fumigatus*	AmB	L/CS	N/A	[68]

**Table 6.** Nanomaterials with antibacterial activities.

Biomaterials	Potential applications	Bacteria	Reference
Cotton/silk fabrics containing reduced graphene oxide (RGO) and Ag/Cu NPs	Antimicrobial protective medical textiles	*P. aeruginosa* *E. coli* *S. aureus*	[69]
Polyvinyl alcohol containing Ag/Cu NPs	Antibacterial contact lenses	*S. aureus* *P. aeruginosa*	[70]
Lysozyme-coated Au NPs in combination with β-lactam	Diabetic wound healing	*S. aureus* *Acinetobacter calcoaceticus* *P. aeruginosa* *E. coli* *Klebsiella pneumonia* *Bacillus subtilis*, *B. cereus*	[71]
Keratin containing Ag NPs	Skin wound healing and tissue recovery	*E. coli* *S. aureus*	[72]
Ag NPs-loaded bacterial cellulose hydrogels	Moist wound-healing hydrogels	*S. aureus* *P. aeruginosa*	[73]

## 3. Nanoparticles (NPs) and their biological compatibility (Table 7)

When NPs come into contact with blood, they may initiate some biological effects, which could be good or bad. Hence, it is important to determine the blood-NPs compatibility before they can be used in humans [74],[75]. A few observations are the following:

- The blood-NPs compatibility depends on the size, structure and formulation of the NPs [74],[76].
- Biopolymeric NPs have been found compatible when used in the treatment of asthma, tuberculosis and lung cancer [77],[78].

**Table 7.** Comparative biocompatibilities of several NPs.

NPs	*In vitro* and *in vivo* toxicity
Dendrimers	No toxic effects [79]
Au NPs	No toxic effects [80]
Carbon nanotubes	No toxic effects [81]
Superparamagnetic Fe3O4 nanoparticles (SPIONs)	No toxic effects [82]
Silica-based NPs	Si NPs cause toxicity to immune cells and tissues. The main mechanisms were pro-inflammatory responses, oxidative stress autophagy and so on. Surface and shape modifications may mitigate the toxicity effects of Si NPs, providing a new way to produce these NMs with less toxic impact [83],[84].
Ag NPs	Induce cell shrinkage, apoptosis [85],[86] Release free radicals and cause DNA damage [87] Immunotoxicity in rats [88],[89] Ag NP-biopolymer showed anti-bacterial activity but no toxic effects on mouse fibroblasts (NIH-3T3), human osteosarcoma cells (MG63) or human hepatocarcinoma cells (HepG2) [90],[91]
Fe3O4-Au NPs	No toxicity was observed in any cell types in culture [92]
Manganese ferrite (MnFe2O4) NPs	Showed biocompatibility at 125 µg/mL or below in 4T1 cells (a murine breast cancer cell line) [93]
Ferrite NPs (Fe3O4, ZnFe3O4 and NiFe3O4)	Showed toxicity against HeLa cell lines at and above 100 µg/mL dosage [94]
TiO2 NPs	These NPs are non-toxic (at <l00 µg/mL) to humans [95]
CaFe2O4 NPs	Showed toxicity in humans at >250 µg/mL concentration [96]

## 4. Nanoparticles (NPs): Encapsulation and biodegradability

Since the accumulation of nanoparticles in the spleen and liver may turn out as toxic, biodegradable NPs (BNPs) should be more appropriate than non-degradable NPs [97]. Other significant factors are the following:

- Nanopolymers are biodegradable and can encapsulate other therapeutic regimens to deliver them to the action site [98].
- Polysaccharides, proteins and some synthetic polymers are the main sources of BNPs.
- Polymersomes (or polymer vesicles) can be used for drug delivery as their coronas and membranes can be modified for biomedical active different groups. Polymersomes are very suitable drug deliver agent for bacterial infection, and cancer therapy, as well.
- Antibacterial polymersomes are divided into three categories:
  1. polymersomes as antibiotic nanocarriers,
  2. intrinsically antibacterial polymersomes and
  3. antibacterial polymersomes with supplementary means, including photothermal and photodynamic therapy.
- Similarly, the anticancer polymersomes are divided into two categories:
  1. Polymersome-based delivery systems, and
  2. Anticancer polymersomes with supplementary means.

In this review, the prospective antibacterial and anticancer polymersomes are discussed.

### 4.1. Selection of polymers and the synthesis of BNPs

The end application is the main criterion for the selection of the polymer, but their size, bio-compatibility, biodegradability and the capability of encapsulation of the drug materials are similarly important factors to be considered [99]. Some of the different biodegradable polymers and their merits for use as BNPs are listed in Table 8.

**Table 8.** Some polymers for the synthesis of BNPs.

Poly lactic-*co*-glycolic acid (PLGA)	Produce biodegradable products, lactic and glycolic acids [100] Generally used in the production of nanovaccines, gene delivery and also the production of protein/peptide-based nanomedicines [100],[101]
Poly lactic acid (PLA)	PLA is biocompatible and biodegradable, breaking down to lactic acid in the body [102]
Gelatin	Gelatin is a polyampholyte and is used in food products and also in medicine [103]
Polycyclic aromatic compounds (PACs)	Upon biodegradation, PACs produce compounds toxic to the central nervous system [104]

## 5. Nanoparticles-mediated microbial targeting strategies

NPs may be considered by the human body as a foreign particle, so macrophages / phagocytic cells can remove them from blood circulation. Therefore, the surfaces of NPs should be modified to allow them to bypass the immune system of the body [105], so they can stay in the vascular system for a longer period of time and may reach their target safely [106]. PEGylation of NPs results in less interaction with phagocytes and being sustained longer in the circulation system [107]. Similarly, tocopherol PEG-1000 succinate can modify NPs, which then in turn exhibit increased adhesion towards tumor cell surfaces [108],[109].

The conventional methods of drug delivery have several limitations, such as poor biodistribution, lack of selectivity and limited effectiveness [110],[111]. Attachment of NPs to the therapeutic drug can make possible site-specific delivery and can reduce any undesirable side effects [112],[113]. Representative clinical trials with small molecule-based targeting have been tabulated elsewhere [114],[115].

### 5.1. Evidence for the attachment of NPs to therapeutic drugs for site-specific delivery

The use of nanotechnology in medicine is mostly for targeted drug delivery and also to reduce toxicity and side effects of the drugs. Until recently, it was not realized that these carrier systems themselves may cause risks to the patient. Therefore, a conceptual understanding of biological responses to nanomaterials is needed to develop [116]–[123].

## 6. Limitations

- The major concern is to maintain the proper size and shape of mono-dispersed NPs with stability during synthesis [124].
- NPs may accumulate in different bio-organs, which may cause problems in normal biological function in the future [125].
- Since NPs may escape the immune challenge of the body, they may cause some sort of inflammation or toxicity [126].
- NPs can generate ROS, which are major contributors of inflammation, oxidative stress and apoptosis [127].
- Still, there are many other disadvantages in using NPs. For example, toxicity, environmental harm and organ damage may be caused by nanoparticles [128].
- Nanoparticles, after a threshold limit, may be toxic in nature and have to be degraded chemically.
- Some identified toxic mechanisms are through the production of ROS, which is cytotoxic, genotoxic, and neurotoxic, also. Those toxic effects of nanoparticles' depends on its type, size, surface area, shape, aspect ratio, surface coating, crystallinity, dissolution and agglomeration properties. Therefore, it is important to consider of any toxic effects of nanoparticles when it is being synthesized [129],[130].

### 6.1. Limited availability and side effects

It has already been demonstrated that many nanoparticles in lab rats have resulted in lung inflammation and blood clotting, and in the human body they could trigger unwanted reactions like damage to cells and organs [131].

- Nanoparticles produce ROS and oxidative stress, which may cause neurodegenerative diseases such as Alzheimer's and Parkinson's diseases [132].
- Uptake of the nanoparticles through the olfactory epithelium can also take place, leading to epithelial cell injury, which can compromise the basic functions of the nose [133].
- Silica exposure causes oxidative stress. At high doses, silica induces membrane damage and cytotoxicity [134].
- Another limitation of using nanotechnology in medicine is its high expense. The use of nanomedicine would increase the cost of health care, which would make its access difficult for the poor [135]. Furthermore, the ethical, social and legal facets of nanomedicine need to be handled tactfully to gain civic backing. Though efforts are being made to increase the understanding of using nanomedicine in living beings, there is still ambiguity surrounding the risks that humans would be exposed to with its use. As a result, the clinical trials involving nanomedicine pose distinctive challenges. The leading ethical issues encompass assessing, managing and communicating the risk during clinical trials. To evade the possibility of public criticism, it becomes imperative to educate the people about the benefits and pitfalls of nanomedicine [136].

## 7. Nanoviricide (NV-387)

**Figure 1.**
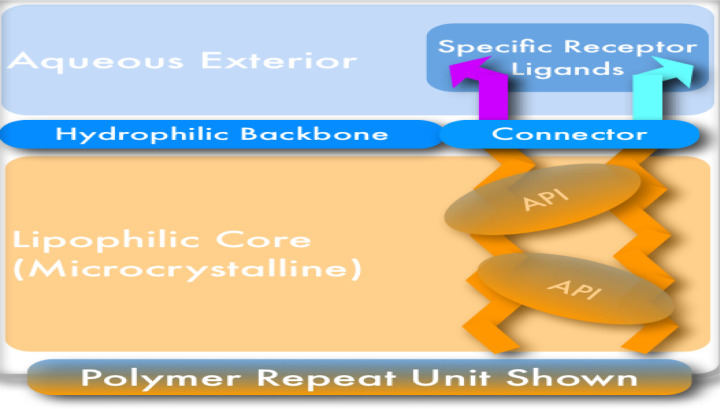
Schematic design of TheraCour NV-387 biopolymer.

A new antiviral regimen could emerge as an antimicrobial. NV-387 is a self-assembling, uniform and tailorable linear homopolymer designed and designated as a TheraCour platform polymer. Here, the monomer is functionalized by attaching polyethylene glycol (PEG) connected covalently with a site-targeting ligand [137] (Figure 1).

This binding results in avidity and that force leads to passive fusion of the virus. Further, being encapsulated, the loaded drug can be released from the polymer backbone in a covalent system immediately [19],[138],[139], TheraCour platform polymer (NV-387) adds further advantages providing an extreme level of tailorability, also:

(A) Different ligands can be chosen for different targets.

(B) By changing the appropriate lipid length and balancing the PEG-monomer chain length, one can balance the hydrophobic/hydrophilic balance of the PEG Polymer. The longer lipid chain would be more suitable for dermal delivery of the drug as a cream or ointment. In contrast, short lipid chains would result more hydrophilic in nature and merely assist in conformational stability and adherence to the cell membrane.

(C) The rate of release of the API can be modified by tailoring the connector, like pH-sensing, or esterase or protease-specific functions, etc.

(D) The polymerization can be controlled within the limits (Flory equation), to provide a desirable clearance characteristics.

NV-387 is a non-crystalline semi-solid, off-white, waxy in texture material (at room temperature). It's theoretical molecular formula is C_104_H_188_N_2_O_44_S_4_. The calculated formula weight of the polymer repeat unit (RU) is 2298.85 g/mol. The degree of polymerization, *“n”*, in P10M2DT (HDA)x (MMSA)y polymer is 8 ± 2 [19]. Pharmaceutical properties, formulations for injection, physical properties, and chemical properties are all available elsewhere [19].

### 7.1. Chemical charateristics of TheraCour biopolymer NV387

NV-387 is a non-crystalline semi-solid, off-white, waxy in texture material (at room temperature). It's theoretical molecular formula is C_104_H_188_N_2_O_44_S_4_. The calculated formula weight of the polymer repeat unit (RU) is 2298.85 g/mol. The degree of polymerization, *“n”*, in P10M2DT (HDA)x (MMSA)y polymer is 8 ± 2 [19].

Pharmaceutical properties, formulations for injection, physical properties, and chemical properties are all available elsewhere [19].

These materials have been shown to be capable of (a) site-directed (address-based) cell or virus targeting, (b) protective active pharmaceutical ingredient (API) encapsulation, (c) direct delivery of such encapsulated APIs into the address-specified cell or virus, (d) tailorable circulation lifetime and (e) sustained delivery characteristics, while at the same time being biocompatibility, non-toxic, non-immunogenic, and biodegradable [137].

### 7.2. Antiviral activity of TheraCour polymer, NV387:

**Figure 2.**
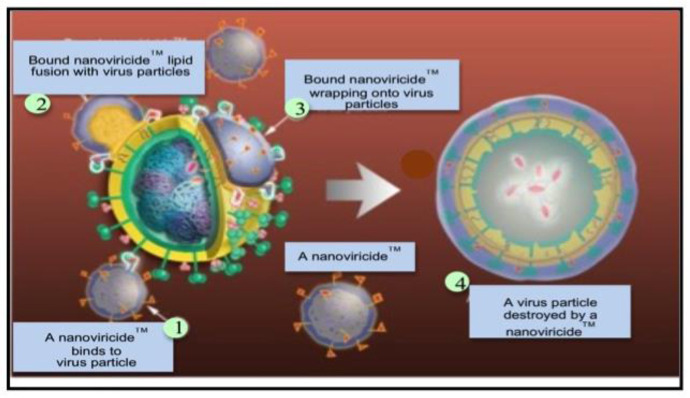
TheraCour Platform Technology based Nanoviricide is a Cell Mimic. A nanoviricide “looks like” a human cell to the virus. A nanoviricide micelle encapsulates the virus particle, even they mutate, and dismantle the virus structure. Step 1: A Nanoviricide™ binds to virus particle; Step 2: Lipid-Lipid fusion of Nanoviricide™ with virus particles; Step 3: Encapsulation of virus particle by Nanoviricide™; Step 4: Nanoviricide™ destroy the virus particle.

In viral diseases, TheraCour platform based nanopolymer, NV-387, is noticeable. The therapeutic principle of NV-387 is based on its unique structure. As we know that the virus envelope carry a lipid membrane derived from the host cell membrane, the TheraCour polymer can attack viruses. Interestingly, no active API is required in this scenario if the ligand is properly chosen for making the biopolymer. Once the virus is attached by the micelle carrying ligands, lipid-lipid mixing essentially pulls the lipid membrane of the virus to the site of the attack and the virus gets dispersed, resulting a naked virus capsid that cannot infect cells (Figure 2) [139]–[143].

### 7.3. Nanoviricide polymeric micelle works against SARS-CoV-2

This model is the most advanced in the antiviral field. In particular, a drug, targeting for SARS-CoV-2 virus, NV-CoV-2 has completed preclinical studies including GLP Safety/Toxicology and is expected to enter human clinical trials soon. Another derivative, NV-CoV-2-R that encapsulates remdesivir within the core of NV-CoV-2 has shown effectiveness significantly surpassing that of the standard remdesivir formulation, which correlates with significantly improved pharmacokinetics of remdesivir *in vivo* in animal model studies. Some uses of TheraCour polymer are the entire drug use chain are shown in Tables 9 and 10.

**Table 9.** TheraCour drug solves problems in the drug use chain.

Vehicle	Administer	Blood Stream	Specific Targeting	Cell Membrane
TheraCour	Injection	Encapsulated	“Nano Velcro Snaking”	Take API Across
Liposomes	Infusion	Unstable	Not Much Success	Partial Effect
Cremophore	Infusion	Unstable	None	Some Effect?
Cydex	Infusion	Full Apart	None	None
PEGylation	Infusion	Stable	None	None
Polydrug	Injection	Stable	None	Depends
Polypeptides	Infusion Injection	Stable	None	None
Dendrimers	Infusion Injection	Toxic	Hard Sphere Few Points	May Take API Across

**Table 10.** TheraCour approach is a unique beneficiaL feature than other nanomedicine approaches.

Vehicle	TheraCour	Dendrimer	PLA/PLGA	Virus Based	Nanoshells, Metalics
Nanoscale Velcro Effect with Wrap-On	Yes	No	No	No	No
Technology Complexity	Simple	Complex	Medium	Complex	Complex
Safety	Safe	No	Medium	No	Medium
Specific Targeting	Yes: Flexible Wrap-ON	Yes: Limited by Hard Bal	No	No	May be
Detection	Yes	Yes	No	No	May be
Extended Release	Yes	May be	Yes	Yes	Accumulate
Controlled Release	Yes	May be	Yes	No	No
Efficacy Improvements	Yes, Very Large	Yes	No (Slow release only)	Yes but infectious	May be

SARS-CoV-2 belongs to a β-family of human coronavirus, which causes the severe lower tract infectious disease called COVID-19 [144]. Throughout the world this pandemic disease virus once evolved in November 2019 is continuously mutating to a new form and infecting people till date. The newer variants (Omicron BA.2) possess greater transmissibility with R0 as 12 [145].

The once effective drugs against SARS-CoV-2, like remdesivir (Gilead), molnupiravir (Merck), and Paxlovid™ (Pfizer) turnout with significant limitations in humans. Molnupiravir is reported as mutagenic and further has poor efficacy. Paxlovid is virostatic and the virus rebounds once the drug is withdrawn. Remdesivir is highly effective *in vitro* studies, however, *in vivo*, its efficacy is not satisfactory at all. This may be due to the instability of Remdesivir in the body circulation system [146],[147].

NV-387 is highly effective in cell cultures against coronavirus hCoV-NL63 which like SARS-CoV-2, binds to the ACE2 cell receptor [140]. hCoV-229E that binds to a different cellular receptor, Aminopeptidase N (APN), also can be inhibited by NV-387, indicating itself as a broad-spectrum anti-coronavirus nanopolymer [20],[147],[148].

### 7.4. Encapsulation of the virus leads to its disintegration

**Figure 3.**
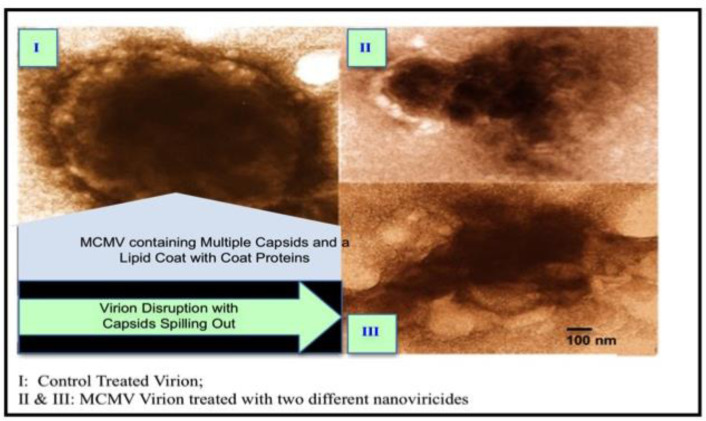
Effects of Two Different Nanoviricides Binding to Murine Cytomegalo virus (MCMV). I: Control virion: MCMV containing multiple capsids and a lipid coat with coat proteins; II & III: MCMV virion treated with two different nanoviricides. Virion disruption with capsids spilling out.

The mechanism of *nanoviricide's* function is shown through electron photomicrographs (Figure 3). In this study, the murine cytomegalovirus (MCMV) was incubated with a *nanoviricide* containing sialic acid as a ligand. The light area at top right corner in Figure 3-II indicates that the lipid coat was deformed due to the binding of nanoviricide micelle in that area. The loss of the viral envelope results the lack of viral glycoproteins required for cellular entry and thus becomes non-infectious. Figure 3-III shows that only virion capsids remain as a result of the attack. We have demonstrated a convincing success of our drug NV-CoV-2-R which is an encapsulated remdesivir into the polymeric micelle (NV-CoV-2), in inhibiting the virus growth in animal models [139],[142].

### 7.5. Safety Studies of NV-387, and NV-CoV-2

NV-387 is a TheraCour biopolymer (API) which on formulation was converted to a drug product against corona virus, and designated as NV-CoV-2. Safety studies on NV-387/NV-CoV-2 indicate that:

- No abnormal respiratory function or in neurobehavioral aspects were notices in all doses of the test compounds was observed in a rat model.
- No change in body temperature after the i.v. administration of NV-CoV-2 in rats.
- Heart rate, blood pressure, cardiac rhythm, and ECG parameters of cynomolgus monkeys were noticed normal after i.v. administration of NV-CoV-2 in them [19].
- Additionally, NV-387/NV-CoV-2, both were non-immunogenic, non-mutagenic, and non-genotoxic in a rat model.

## 8. Discussion and conclusions

The use of nanomaterials has been increasing, with concerns about drug-nanomaterial stability, biocompatibility and biodegradability; and there is interest in control and tailored payload release of the drug, without any side effects, and improving patient compliance [149],[150]. With these concerns, recently, nucleic acid-based cross-linkers, as they are able to self-assemble into a stable 3-dimensional structures, have gained much attention [149]–[151]. In addition, nucleic acids can act as a targeting agent through engineered aptamer and drug payload carriers. They also have shown the ability to control the release of proteins [152]–[155]. Owing to these versatile characteristics, it is expected that nucleic acid-based hydrogels will be an important regimen in the future for targeted drug release.

Treatment of infectious disease with antibiotics becomes a challenge when the organisms evolve drug resistance. Therefore, discovery of methods of treatment and/or therapeutic regimen warrants great priority. Nanotechnology offers an innovative advance in NP-based bio-imaging, which can be used for early detection, diagnosis and treatment of many diseases, especially those that are caused by drug-resistant microorganisms. Nanoparticles have been shown, due to their unique size, shape, charge and surface area, to possess unique activity against different microbial infections. In addition, NPs find their other uses in drug delivery, gene delivery and targeted therapy of various diseases including cancer.

The development of nanotechnology for the synthesis of NPs/nanocomposites can be used to treat various diseases which are difficult to treat with the conventional approaches. However, the limitations and health risks that are associated with these nano-sized particles should not be ignored. Nowadays, in many cases, nanotherapy along with the conventional antibiotic therapy is used to overcome microbial resistance. NPs/nanocomposites may resolve difficulties in managing complicated diseases. However, safety and efficacy issues of NPs are now the main concern before their use in humans.
